# Incorporation of Patient and Public Involvement in Statistical Methodology Research: Summary of Workshop Proceedings

**DOI:** 10.1002/sim.70159

**Published:** 2025-07-15

**Authors:** Aiden Smith, Hannah Worboys, Samina Begum, Derrick Bennett, Jonathan Broomfield, Suzie Cro, Laura Evans‐Hill, Justin Greenwood, Ania Henley, Mary Mancini, Kara‐Louise Royle, Helen Saul, Jamie Sergeant, Derek Stewart, Freya Tyrer, James Wason, Christopher Yau, Laura J. Gray

**Affiliations:** ^1^ Department of Population Health Sciences University of Leicester Leicester UK; ^2^ Public Contributor; ^3^ Department of Population Health University of Oxford Nuffield Oxford UK; ^4^ School of Public Health Imperial College London London UK; ^5^ Nifty Fox Creative Barnsley UK; ^6^ Clinical Trials Research Unit University of Leeds Leeds UK; ^7^ National Institute for Health and Care Research London UK; ^8^ School of Health Sciences University of Manchester Manchester UK; ^9^ Diabetes Research Centre University of Leicester Leicester UK; ^10^ Population Health Sciences Institute Newcastle University Newcastle UK; ^11^ Department of Women's and Reproductive Health University of Oxford Nuffield Oxford UK

**Keywords:** methods research, patient and public involvement, patient engagement, statistical methodology

## Abstract

Patient and Public Involvement (PPI) is well‐established in applied health research but remains under utilised in statistical methodology research due to perceived irrelevance and communication challenges. This paper summarises a one‐day workshop held in February 2024 in Leicester, organised by the University of Leicester and the NIHR Statistics Group, aimed at addressing barriers to meaningful PPI in statistical methodology. The workshop brought together statisticians and experienced public contributors to discuss strategies, share case studies, and offer practical guidance on conducting effective PPI. Key barriers identified included: (1) uncertainty about the relevance of PPI in methodology‐focused research; (2) public contributors' anxiety over mathematical complexity; and (3) mismatched expectations due to different backgrounds in applied versus methodological research. Case studies showcased how PPI led to improved model structures, identification of data issues, and enhanced study materials. The importance of communication was a recurrent theme, with recommendations including use of plain English, regular updates, and visual storytelling tools. Feedback from attendees indicated increased confidence and motivation to engage in PPI. Public contributors emphasised the need for respectful, non‐patronising interactions and flexible roles within projects. Recommendations include managing expectations, enhancing accessibility, co‐developing materials, and fostering diversity among contributors. This paper highlights the need for tailored strategies to integrate PPI into statistical methodology, including the development of resources (e.g., glossaries, animations) and further case study collection. Future work will focus on expanding these resources, addressing challenges of equity and inclusion, and supporting PPI in complex methodological areas like simulation and model development.

## 1 Introduction

Patient and Public Involvement (PPI) is defined by the National Institute for Health and Care Research (NIHR) as research being carried out “with” or “by” members of the public rather than “to,” “about,” or “for” them [1]. The benefits of PPI to healthcare research are well‐recognized, and include increasing quality, relevance, and impact through expert knowledge of specific health conditions or health system interactions [2, 3]. Further benefits have also been reported for public contributors through enhancement of personal development, knowledge, and satisfaction [4]. Whilst PPI is embedded in applied (people‐based) healthcare research, it can be more challenging for statistical methodology research. Statistical methodology research involves improving, developing, comparing, or evaluating statistical methods, such as mathematical models and techniques. Our definition of statistical methodology research refers specifically to statistical methods which are to be used in studies related to health and social care. For these types of studies, difficult decisions need to be made on how to carry out PPI such that contributions are meaningful and not simply tokenistic to meet funding requirements [5].

This article summarizes the presentations and discussions from a 1‐day workshop about PPI in statistical methodology research organized by the University of Leicester and the Improving Statistical Literacy working group of the NIHR Statistics Group, which was held in Leicester on 26 February 2024. Here, we give an overview of the workshop discussions, lessons learned, and recommendations for the future application of PPI in statistical methodology research.

## 2 Methods

Invitations for expressions of interest to attend the event were sent to statisticians and epidemiologists via the authors' professional networks, social media, mailing lists, and institutions. In addition, methodologists were able to request attendance of public contributors they had worked with (although no public contributors attended through this mechanism). In total, 124 people expressed an interest in attending. Of these, 62 people were selected and attended the workshop—all were statisticians. We selected attendees from a range of institutions and career stages to maximize diversity. The 62 statisticians came from 35 academic institutions, spanning all career stages from PhD students to Professors.

In addition to the attendees, nine statisticians and five public contributors were invited and agreed to present at the workshop based on their contributions to previous statistical methodological research.

### 2.1 Aims

The aims of the workshop were to identify barriers to conducting PPI to inform statistical methodology research, give case studies of impactful PPI for statistical methodology research, and deliver practical advice for conducting PPI. The aims were met through presentations and open discussions.

## 3 Summary of Proceedings

The workshop constituted presentations from researchers and public contributors, with time allowed for open discussions. Additional sessions were given on writing a plain English summary (Helen Saul, NIHR) and story‐telling (interactive session, facilitated by Laura Evans‐Hill, Nifty Fox Creative). The summary of proceedings was as follows:

- “Welcome and Introduction to the Improving Statistical Literacy Working Group of the NIHR Statistics Group”—Derrick Bennett & Jamie Sergeant
- “Introduction to PPI for Statistical Methodology Research”—Laura Gray & Samina Begum
- “Public Contributors' Perspectives”
  ∘ “Introduction to Public Contributors & How They Became Involved in PPI for Statistical Methodology Research”—Ania Henley, Derek Stewart, Samina Begum, Mary Mancini & Justin Greenwood
  ∘ “Public Contributor Experiences of PPI Involvement in Statistical Methodology Projects”—Ania Henley, Suzie Cro & Derek Stewart
- “Case Studies Highlighting Impactful PPI”—Jonathan Broomfield, Freya Tyrer, Kara‐Louise Royle, Christopher Yau & James Wason
- “How to Write a Good Plain English Summary”—Helen Saul, Medical Journalist
- “Interactive Session on Storytelling and Getting Your Message Across”—Laura Evans‐Hill, Founder and Director of Nifty Fox Creative
- “Summation of the Event & Feedback”—Derrick Bennett, Jamie Sergeant & Laura Gray

An overview of the main themes from the discussions are outlined below. Copies of the presentations can be accessed at https://leicesterbrc.nihr.ac.uk/ppismart/ppiestatsleicester/.

## 4 Main Findings

### 4.1 Barriers to Conducting PPI to Inform Statistical Methodology Research

#### 4.1.1 PPI Not Judged to Be Relevant in Statistical Methodology Research

In previous survey‐based research, a minority of methodologists have questioned the involvement of PPI as “statistical methods shouldn't alter depending on what patients think…” [6]. Around half of the attendees at the workshop had not undertaken PPI previously in statistical methodology research (21/43 respondents from feedback questionnaire [49%]). Despite this, the researchers in attendance reported that they wanted to understand how to effectively engage members of the public in their research, 55/58 respondents from the feedback questionnaire felt the workshop had improved their confidence in conducting PPI.

#### 4.1.2 Anxiety Over Lack of Mathematics Skills (On the Part of Public Contributors)

During the workshop, public contributors expressed that prior to getting involved with PPI projects they had anxiety over their lack of mathematical knowledge and not understanding the terminology used in discussions. Specifically, the use of acronyms, jargon, and complex statistical terms that were not explained were not always understood. This was further compounded by language barriers. Although it was recognized that researchers should explain studies in simple terms [7], lack of effective communication was a significant barrier leading to public contributors feeling isolated and disenfranchised.

#### 4.1.3 Imbalance Between Public Contributors' Expectations and Reality

The public contributors presented on their involvement in research, which had typically started in applied healthcare research studies where they had been asked to provide feedback on their own lived experience of specific health conditions. When they became involved in statistical methodology research, they needed to reframe their expectations to incorporate broader perspectives across different health conditions. That said, one public attendee did comment that the COVID pandemic had prompted them to consider the portrayal and communication of statistics, which had contributed to their interest in this area. Another commented that their work experiences in relation to the inefficient use of data had motivated their involvement.

### 4.2 Case Studies of Impactful PPI for Statistical Methodology Research

A joint presentation by a public contributor and researcher (Ania Henley, Suzie Cro) highlighted effective communication through the co‐development of a tool to explain and interpret “estimands” for members of the public [8]. Five case studies were also presented by statisticians to highlight successful interactions with public contributors, outlined in Table 1.

**TABLE 1:** Case studies of successful PPI in statistical methodology research.

Title of project	Name of Researcher	Aim of project	Inclusion of PPI	Impact of including PPI
(1) Natural history modeling in rare disease	Jonathan Broomfield, University of Leicester	To build a natural history model, the typical progression of a disease over time, for patients with Duchenne Muscular Dystrophy (DMD). The model has several health states which break down the disease into different stages. Patients move from one health state to another as the disease progresses.	Prior to this project, patients were categorized into two stages: ambulatory and non‐ambulatory (of which there can be several health states). PPI members identified an additional health state to be added to the model, between ambulatory and non‐ambulatory.	The analysis of this additional state shows that patients had an improved quality of life and lower costs in the transfer state vs. the non‐ambulatory state. Without PPI the results would be biased, underestimating the quality of life and overestimating direct treatment costs in patient with DMD.
(2) Immortal time bias	Freya Tyrer, University of Leicester	To look at GP data on differences in life expectancy for people with/without learning difficulties.	PPI members were asked in an interview about their health and experiences within the health care system. PPI members raised issues about time spent and difficulty getting a diagnosis, as well as finding that when moving between primary and secondary care, health care records weren't up to date.	The comments from the PPI meeting helped the researcher find an anomaly in the data which, when they accounted for this, made the results look much more credible and expected. Without some of the comments related to delayed and missing records, the researchers would not have thought to look at the patterns of diagnoses or found the spike in records that explain the anomaly.
(3) Overall survival in cancer trials	Kara Louise Royle, University of Leeds	To obtain a consensus from stakeholders (including patients and the public) about how overall survival should be analyzed in cancer clinical trials, when trial participants receive subsequent treatment lines during trial follow‐up.	PPI members supported the grant application, co‐designed the PPI plan, questionnaires, and participant materials for the study and facilitated a focus group meeting.	PPI involvement ensured that the consensus exercise was accessible to patient/public participants when answering questions about statistical concepts such as data collection and statistical assumptions. The researcher also recorded very high completion rates of the questionnaires and attribute this to involving PPI members from the start.
(4) Innovative trial design	James Wason, Newcastle University	This project was an application for an NIHR Research Professorship. The project focused on innovative trial design, and how to tailor trials to chronic inflammatory conditions.	PPI helped form key parts of the research proposal itself, then during the study PPI members gave input on what aspects of innovative designs could be improved. PPI members felt that more trials in general was the most important factor, as trials in this disease area were rare.	PPI input facilitated work on methodological developments for trial design in the specific disease area. It led to adaptive trial designs, improved statistical efficiency of analysis where diseases are rare, and ensured much clearer language was used—also leading to the creation of visualizations.
(5) Data science in cancer research	Christopher Yau, University of Oxford	To improve the understanding of how treatments for ovarian cancer work, and whether new therapies can be developed using advanced biotechnologies and artificial intelligence. The project investigated the development of predictive models for the probability of successful treatment, given a patients cancer profile. A key part of the project was identifying how non‐statisticians interpret outputs from these prediction models.	A series of PPI events were hosted in conjunction with Ovarian Cancer Action, and a report was produced to convey how the project was changed as a result. Using a survey to assess mathematical literacy in the general public, findings showed a low level of general understanding which led to questions regarding how best to disseminate mathematical concepts to the general population.	Involving PPI in this project made the research team re‐prioritize their research aims to ensure the outcomes of the research could be communicated effectively to non‐statisticians.

In Case Study 1, PPI members helped the researchers by identifying an additional health state in a natural history model, leading to a significant change in the economic analyses [9]. Case Study 2 involved discussions with carers and people with learning disabilities, which helped the researcher to identify an anomaly in the recording of life‐long conditions in primary healthcare data, leading to immortal time bias. This led to revising the methodological approach, which can now be applied in other settings [10]. In Case Study 3, PPI members contributed to discussions around survival analyses, ensuring study materials were accessible, this led to very high completion rates, which the authors believe to be directly attributable to PPI involvement. Case Study 4 provided the reflections of a statistician applying for an NIHR Research Professorship, and how PPI helped shape their application on trial design for rare diseases. Case Study 5 offered insight into how personalized treatment decisions for ovarian cancer might be refined and effectively communicated through the incorporation of PPI. Slides for the talks outlined here can be found through the link to the workshop in the “Useful Links” list in Supporting Information 2. In the feedback from the workshop, some respondents felt that the research in the case studies and examples was too applied, and they would have liked to have seen examples showcasing PPI input into research further away from the applied end of the spectrum. When preparing for the workshop, we asked researchers to provide case studies of their own involvement activities. Many were towards the applied end of statistical methodology research, which highlights that PPI in statistical methodology research is currently limited. A recent survey of statistical methodologists assessing their involvement in PPI in statistical methodology research found that only 20% of respondents had conducted PPI during a methodology project. A further 40% of respondents reported that they undertook PPI to inform a grant application [6]. Sharing successful experiences of PPI for complex methodologies, such as model development and simulation studies, would help to break down many of the barriers to PPI in statistical methodology research, particularly for those who judge that their work is too technical to communicate to non‐specialized audiences or who lack confidence to carry out PPI.

Another presenter (Suzie Cro) who formed a public involvement group that met several times throughout a 3‐year project found benefits in co‐chairing the group with an experienced public contributor. The public partner co‐chair reviewed meeting materials prior to meeting occurrences, which enabled meetings to run smoothly and ensured other public contributors were involved and engaged in meetings. This may be particularly valuable to researchers who are new to public involvement.

The case studies and joint researcher/public contributor presentation highlighted lessons that could be learned from the process. This included better communication with public contributors at the earlier stages of the research, providing accessible information in advance, and asking questions about the data sources used (e.g., primary care health data). One of the presenters pointed out a struggle between whether to allow discussions that were not directly relevant to the research to continue (“to be polite”) or bring the conversation back to the prepared questions. Of course, this conflict is not exclusive to statistical methodology PPI work. From a researchers perspective, this can stem from knowing that time with PPI groups is limited and the desire to get as much from the sessions as possible can see researchers hastening to keep the conversation very directly related to the project. However, by allowing these less‐relevant discussions to imbue themselves into the sessions, the researcher can facilitate team‐building and relationship development among the public contributors, which may be beneficial in the long‐term.

### 4.3 Practical Advice for Conducting PPI for Statistical Methodology Research

An overarching theme throughout the workshop was the use of communication strategies to ensure that public contributors were not isolated or disengaged. Strategies included giving contributors a recap of information discussed at prior meetings either verbally or using newsletters before commencing new PPI activities. Regular communication in between meetings was also perceived to be important to maintain engagement.

The final event at the workshop was a story‐telling session which highlighted the importance of creative communication strategies, hosted by Nifty Fox Creative. The need to make research more accessible has led to a change in direction from classic journal articles that are text, table, and graph‐heavy towards more creative ways of explaining the research. Such approaches have been found to be more effective in reaching diverse audiences [11], improving relationships between researchers and the community [12], and facilitating the creation of high‐quality research [5]. Visual storytelling is the art of using pictures (visual abstracts, animations, infographics, and interactive websites) to translate complicated ideas into simple narratives. Visual stories are more accessible and easily understood than traditional journal articles as people tend to process visuals more easily than text [13]. Journal articles with a visual abstract also typically have more views than those without [14]. Disregarding the input of public contributors is a missed opportunity for visual storytelling as they are uniquely placed to provide feedback on the relevance and clarity of the visual methods adopted. This is particularly true for research of statistical methodology, given its technical complexities.

Public contributors also highlighted the importance of respect, and researchers not being patronizing. Furthermore, it was deemed important by the public contributors that an environment was cultivated in which they felt comfortable criticizing or critiquing the research project in question freely. Similarly, public contributors should be able to dictate the level in which they feel they can impact a project, and if they feel that their input is not useful, they can withdraw without feeling pressure to remain involved and provide tokenistic input. It is important to cost adequately for public contributor time, including time in between meetings to review work. Public contributors also welcomed offers of co‐authorship for resulting publications; however, it should not be assumed that all will want this.

### 4.4 Plain English Summaries

A key presentation from the workshop was on how to write an effective plain English summary. Although this is not a topic which is specific to statistical methodology research; our public contributors felt this was an important topic to cover.

This is a pre‐requisite of many funding bodies when submitting grant applications—and is often read by all parties involved in the review process. Plain English summaries are most effective when they meet the needs of the audience, do not include unnecessary words, and present findings clearly. The involvement of public contributors in this process is crucial for effective communication to non‐scientific audiences and also provides reassurance for public reviewers who are involved in making decisions about whether to fund the research project. It is important to acknowledge that when writing a plain English summary that public contributor input should be consistent throughout. Many researchers and clinicians overestimate their capacity to write in truly plain English, and therefore these summaries should be reviewed by a public contributor (ideally multiple times) before being submitted. Key considerations for the researcher when writing a plain English summary include: (i) highlighting the importance of the study and what the reader needs to know; (ii) avoiding acronyms/initialisms and repetition; (iii) using the active tense; and (iv) use of bullet points/visuals.

## 5 Feedback From Workshop Participants

Following the conclusion of the workshop, attendees were invited to complete a feedback questionnaire. Feedback was obtained via a questionnaire that was given to all attendees immediately via a QR code; providing feedback was optional. The data collected included: the role and career stage of the individual, their experience of using PPI to date, their feelings about the workshop itself, whether they felt more confident conducting PPI in methodological research having undertaken the workshop, and providing free text options for further comment. None of the questions were compulsory. The feedback questionnaire can be seen in full in Supporting Information 1.

Feedback was provided by 63 attendees (one public contributor and 62 statisticians).

The feedback received from attendees from the workshop was overwhelmingly positive, with a mean satisfaction of 8.9/10 (62 respondents) and attendees reporting feeling more confident about doing PPI (7.8/10; 60 respondents) and developing new skills (8.1/10; 60 respondents).

Some interesting insights were found from the participant feedback forms that captured what participants had taken from the workshop. One participants takeaway related to previous feelings of needing to have fully fleshed out questions for your PPI group, but the workshop has demonstrated that “you don't necessarily need to have the questions to go to the PPI group with before you meet.” Another comment centered around the new understanding that “PPI takes time and preparation” and shouldn't just be tokenistic. One participant provided feedback that researchers “should not go into PPI assuming what the public contributors want. Have an open mind towards it.” The workshop helped researchers understand ways in which PPI can be useful to methodology research and ensure public contributors remain engaged with the projects.

The complete data from the feedback forms is provided in Supporting Information 3.

## 6 Discussion and Recommendations

This article summarizes the proceedings of a 1‐day workshop about PPI in statistical methodology research, highlighting the barriers to PPI, some examples of successful PPI activities and suggestions on how to improve communication. Overall, the interest shown in the workshop (with maximum capacity of attendees reached) emphasized that statistical methodology in PPI research is important to statisticians, themselves. However, the barriers discussed also highlight the significant challenges to “meaningful” involvement that could not be fully teased out in this workshop setting.

Following the workshop, we propose some broad recommendations for involving members of the public in statistical methodology research:

- **Manage expectations** at the outset. It is natural for public contributors to fear the “scary maths” aspects of statistical methodology research or to not understand how statistical methodology research differs from applied health research. Making sure that public contributors understand the type of research you are conducting is important for all research areas, but perhaps even more so for statistical methodology research. One proposed resource for this is a co‐developed animation by the University of Leicester and PPI‐SMART group to describe statistical methodology research and the importance of PPI [5].
- **Take the time to communicate** with public contributors. Keep language simple, clear, and unambiguous. It is more important for public contributors to understand the concept of what the research is trying to do and its possible benefits than to fully understand the details and statistical complexities.
- **Keep public contributors engaged**. Give regular updates on what is happening in the research and how previous comments have been addressed.
- **Involve public contributors in the plain English summary**. Make sure technical elements are explained clearly and concisely, which may be more challenging and take longer to do than other types of research. A good plain English summary will always improve the likelihood of the research being funded/read; for a statistical methodology application, this summary might also be important for other non‐statistician panel members.
- **Use visual storytelling devices to communicate the research and findings**. A consistent finding across case studies is that analogies and storytelling can help break down complex statistical ideas in an accessible way.

One further recommendation that was not discussed extensively at the workshop is to consider diversity when carrying out PPI in statistical methodology research. Efforts to widen the diversity of public contributors are likely to also widen the range of responses and make the research more relevant and understandable to all. There are likely to be additional barriers when recruiting public members from diverse communities to this type of research. The PPI‐SMART team has been conducting research focusing on equality, diversity, and inclusion in statistical methodology research [15]. This work includes adapting the NIHR EDI Toolkit specifically for methodological projects, such that resources are available to researchers to make EDI implementation easier.

There are many advantages of utilizing public contributors with previous PPI experience. Those who regularly act as public contributors for research projects can be brought up to speed quickly and spend more time providing feedback on the project. However, it may also be important to reach a completely new audience, particularly where the aim is to disseminate to the general public who are new to statistical methods. Decisions on who to involve, ideally with both experienced and inexperienced public contributors, are important at the outset. Previous work critically explores how researchers perceive and manage the knowledge and experience levels of public contributors in applied health research [16]. They make distinctions about the two levels of experience for different types of public contributors and subsequent treatment of such experience levels from researchers. The authors argue that both types of knowledge (expert and naïve) are valuable and necessary, and the real issue lies not in the contributors' experience levels but in how researchers choose to engage with them. Interested readers are encouraged to read this paper and reflect on their own experiences.

## 7 Limitations and Future Work

These findings summarize a single workshop, and the voices of public contributors were limited to those who presented and had experience of statistical methodological work. Moreover, from the workshop's feedback questionnaire, it was noted that there were only a small number of case studies from academics conducting PPI in statistical methodology research. PPI was not conducted widely in this area, and all of the case studies had applications to real‐world settings. We plan to continue collecting case studies as more PPI is conducted in this area to extend our catalogue of examples.

The barriers to conducting PPI work that we identified could only be discussed briefly in this workshop setting. We recognize that there are tensions to involving public partners, particularly where deadlines are tight and adequate time and resources are needed for effective communication. We also acknowledge the need to constantly reflect on the involvement, whether it is meaningful, and whether it is the public partner or the researcher who benefits most from the involvement. We would welcome a future workshop to focus on different ways to address the barriers that we have identified to increase the confidence of both researchers and public contributors. Further development work by the PPI‐SMART group involves the development of a plain English glossary of fundamental mathematical and statistical terms to facilitate understanding of complex terminology. We would also like to see concrete examples of PPI used to inform more complex aspects of statistical methodology research, such as simulation studies and model development. The use of new or existing frameworks to inform PPI in statistical methodology research is also indicated. For researchers, we have provided a list of useful resources in Supporting Information 2, which contains resources to utilize in PPI sessions (an animation and glossary for describing statistical methodology and associated terminology), slides from the workshop itself, the NIHR EDI toolkit, and others.

## Conflicts of Interest

The authors declare no conflicts of interest.

## Supporting information


**Data S1:** Supporting Information.

**Data S2:** Supporting Information.

**Data S3:** Supporting Information.

## Data Availability

Data sharing not applicable to this article as no datasets were generated or analysed during the current study.
