# Supplementary material for: Incorporation of Patient and Public Involvement in Statistical Methodology Research: Summary of Workshop Proceedings
Source: Stat Med. 2025 Jul 15;44(15-17):e70159. doi: 10.1002/sim.70159 (PMC12261386; doi:10.1002/sim.70159)
Supplement: Supplementary file 1 — Data S1: Supporting Information. [file SIM-44-0-s002.docx]

**Feedback form**

Q1: Are you a public contributor?

- Yes
- No

**Questions for researchers only**

Q1a: If No then ask – what is your career stage (please select the closet to your current role)?

- Research Assistant (pre-doc or PhD student)
- Research Associate (post-doc)
- Research Fellow / Lecturer
- Senior Research Fellow / Associate Professor
- Professor

Q1b: If No then ask – which of these best describes your PPI experience to date?

- I haven’t undertaken any PPI
- I have undertaken PPI to inform methodological research
- I have undertaken PPI to inform applied research
- I have undertaken PPI to inform applied and methodological research

**Questions for public contributors only**

Q1a: If yes then ask – which of these best describes your PPI experience to date?

- I haven’t provided any PPI
- I have provided PPI to inform methodological research
- I have provided PPI to inform healthcare research
- I have provided PPI to inform healthcare and methodological research

**Questions for all**

Q2: How satisfied were you with the workshop on a scale of 1-10 (1=least satisfied, 10=most satisfied)?

- Scale 1-10

Q3: What did you like most about the workshop?

- Free text

Q4: What did you like least about the workshop?

- Free text

Q5: How much do you feel the workshop has improved your confidence in undertaking PPI for methodological research on a scale of 1-10 (1=least improvement, 10=most improvement)?

- Scale 1-10

Q6: How much do you feel the workshop has given you new skills in undertaking PPI for methodological research on a scale of 1-10 (1=no new skills, 10=lots of new skills)?

- Scale 1-10

Q7: What was your biggest takeaway from this event?

- Free text

Q8: Would you attend another event like this in the future?

- Yes
- No

Q9: Would you recommend attending this workshop to your colleagues?

- Yes
- No

Q10: Any other comments you would like to leave

- Free text
