# Supplementary material for: Incorporation of Patient and Public Involvement in Statistical Methodology Research: Summary of Workshop Proceedings
Source: Stat Med. 2025 Jul 15;44(15-17):e70159. doi: 10.1002/sim.70159 (PMC12261386; doi:10.1002/sim.70159)
Supplement: Supplementary file 2 — Data S2: Supporting Information. [file SIM-44-0-s003.docx]

## Supplementary Materials 2: Useful Resources

An animation explaining statistical methodology research to the lay person - <https://www.learningforinvolvement.org.uk/content/resource/what-is-statistical-methodology-research-and-why-is-ppie-input-important/>

NIHR Public Information Pack - <https://www.nihr.ac.uk/documents/public-information-pack-pip-how-to-get-involved-in-nhs-public-health-and-social-care-research/27388>

NIHR EDI Toolkit - <https://www.rdsresources.org.uk/edi-toolkit?tags=EDI>

PPI-SMART Website - <https://leicesterbrc.nihr.ac.uk/ppismart/>

NIHR Strengthening EDI and PPIE - <https://www.nihr.ac.uk/documents/strengthening-our-commitment-to-equality-diversity-inclusion-and-patient-and-public-involvement-and-engagement-ppie/24697>

Workshop Slides - <https://leicesterbrc.nihr.ac.uk/ppismart/ppiestatsleicester/>

Plain English glossary of terms used in statistical methodology research - <https://leicesterbrc.nihr.ac.uk/ppismart/ppismart-definitions/>

The PPI-SMART team X (Twitter) page - <https://x.com/Stats_PPIE>
